# Coronene and Phthalocyanine Trapping Efficiency of a Two-Dimensional Kagomé Host-Nanoarchitecture

**DOI:** 10.3390/nano12050775

**Published:** 2022-02-25

**Authors:** Yi Wang, Xinrui Miao, Wenli Deng, Romain Brisse, Bruno Jousselme, Fabien Silly

**Affiliations:** 1 School of Materials Science and Engineering, South China University of Technology, Guangzhou 510640, China; 201810103602@mail.scut.edu.cn (Y.W.); wldeng@scut.edu.cn (W.D.); 2 Université Paris-Saclay, CEA, CNRS, NIMBE, LICSEN, F-91191 Gif sur Yvette, France; romain.brisse@agencerecherche.fr (R.B.); brunoi.jousselme@cea.fr (B.J.); 3 Université Paris-Saclay, CEA, CNRS, SPEC, TITANS, F-91191 Gif sur Yvette, France

**Keywords:** molecular self-assembly, guest-host structures, 2D material, scanning tunneling microscopy, intermolecular interactions, hydrogen bonds

## Abstract

The trapping of coronene and zinc phthalocyanine (ZnPc) molecules at low concentration by a two-dimensional self-assembled nanoarchitecture of a push–pull dye is investigated using scanning tunneling microscopy (STM) at the liquid–solid interface. The push–pull molecules adopt an L-shaped conformation and self-assemble on a graphite surface into a hydrogen-bonded Kagomé network with porous hexagonal cavities. This porous host-structure is used to trap coronene and ZnPc guest molecules. STM images reveal that only 11% of the Kagomé network cavities are filled with coronene molecules. In addition, these guest molecules are not locked in the host-network and are desorbing from the surface. In contrast, STM results reveal that the occupancy of the Kagomé cavities by ZnPc evolves linearly with time until 95% are occupied and that the host structure cavities are all occupied after few hours.

## 1. Introduction

Porous materials and nanoarchitectures are of scientific and technological interest because of their ability to interact with foreign nanospecies throughout specific functional sites located in their internal structure or surface [1,2,3,4]. Research has been devoted to assessing the structure property correlations and interactions between the host-structure and guest-species. In this respect, the construction of organic nanoarchitectures and thus, porous host-structure through molecular self-assembly [5,6,7] is especially appealing [8,9,10]. Selective and directional intermolecular binding by halogen bonds [11,12,13,14,15,16,17] as well as hydrogen bonds [5,18,19,20,21,22,23,24,25] has been successfully exploited to govern molecular self-assembly. Even multi-component organic two-dimensional nanoarchitectures have been successfully achieved [26,27,28]. Multicomponent organic nanoarchitectures have also been engineered by the formation of guest-host structures, where foreign molecules are trapped inside the cavities of a porous 2D network [29,30]. The size and shape of the trapped molecules, as well as those of the host structure cavities, are key parameters, which drastically affect the efficiency of guest-molecule trapping by the host structure [31,32]. Scanning tunneling microscopy (STM) is a powerful tool with submolecular resolution to not only characterize molecular assembly but also probe various dynamic processes appearing during the formation of a guest-host structure. For example, evidence for single-molecule adsorption/desorption events have been identified in sequential STM images [33].

We recently successfully engineered a porous two-dimensional (2D) Kagomé host-nanoarchitecture through the self-assembly of “push–pull” dyes and used this structure to trap round coronene molecules at high concentration [34]. However, the ability of a host structure has to be assessed at low guest-molecule concentration to determine its efficiency and stability. It is also unclear whether this host structure is versatile enough to trap larger functionalized cross-shaped complexes, such as those of phthalocyanines [35,36,37]. Phthalocyanines are especially appealing organic compounds and have attracted considerable interest in recent years due to their potential applications in organic transistors [38], solar cells [39] and information storage systems [40].

In this paper, we investigate the molecular trapping efficiency at low-concentration of two-dimensional host-nanoarchitecture composed of push–pull dye. STM is used to locally assess if coronene and ZnPc guest molecules can be trapped into the cavities of the dye host structure.

## 2. Materials and Methods

**Figure 1:**
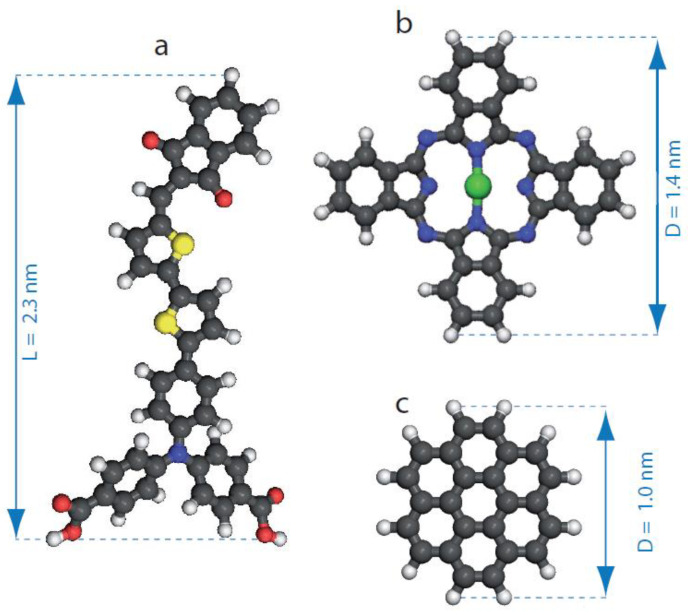
(**a**) Scheme of *N*,*N*-di(4-benzoicacid)-4-(5′-[(indan-1,3-dion-2-ylidene)methyl]-2,2′-bithien-5-yl)-phenylamine molecule(C_38_H_24_N_6_O_6_S_2_). (**b**) Scheme of zinc phthalocyanine (ZnPc), C_32_H_16_N_8_Zn. (**c**) Scheme of coronene molecule, C_24_H_12_. Carbon atoms are represented in gray, hydrogen atoms in white, oxygen atoms in red, nitrogen atoms in blue, sulfur atoms in yellow and zinc atom in green, respectively.

**Materials and sample preparation:** The structure of the push–pull dye, *N*,*N*-di(4-benzoic acid)-4-(5′-[(indan-1,3-dion-2-ylidene)methyl-2,2′-bithien-5-yl)-phenylamine (compound-1, C_38_H_24_N_6_O_6_S_2_), is depicted in Figure 1a. This molecule is composed of an indandione head, a bithiophene backbone and a triphenylamine tail. Carboxylic groups have been grafted onto the triphenylamine group to promote intermolecular double hydrogen-bonds (O–H···O). This interaction has been successfully used to stabilize the formation of porous as well as compact nanoarchitectures on surfaces [41,42,43,44,45,46,47]. The compound-1 was synthesized according to the procedure described in ref. [48]. First, a 1.0 × 10^−3^ M solution of compound-1 in 1-octanoic acid was prepared and it was then ultrasonicated for 20 min in a centrifuge tube. Finally, the solution was diluted to reach the target value of 10^−5^–10^−6^ M. The structure of zinc phthalocyanine (ZnPc, C_32_H_16_N_8_Zn) is presented in Figure 1b. This cross-shaped molecule has a Zn atom at its center and its diameter is 1.4 nm. Solution of ZnPc at a low concentration (2.0 × 10^−6^ M), also in 1-octanoic acid, was prepared. The structure of the coronene molecule (C_24_H_12_) is presented in Figure 1c, which has a round shape and the diameter of ∼1.0 nm. Solution of coronene at a low concentration (2.0 × 10^−6^ M), also in 1-octanoic acid, was prepared.

**STM imaging:** After obtaining STM images of assembled adlayer of compound-1 at the solid–liquid interface, a drop of ZnPc or coronene solution was deposited on the same highly ordered pyrolytic graphite (HOPG) surface (Bruker, Billerica, MA, USA, quality ZYB grade). STM measurements were preformed straight after guest molecule deposition. A physical monolayer formed spontaneously. STM imaging of the samples was performed at the liquid/solid interface using a Nanoscope IIIa Multimode SPM (Bruker, Billerica, MA, USA) scanning tunneling microscope. Cut Pt/Ir tips were used to obtain constant current images at room temperature with a bias voltage applied to the sample. Positive tunneling bias therefore corresponds to tunnelling into the sample empty states, whereas negative bias corresponds to tunnelling from the sample filled states. STM images were processed and analyzed using the application FabViewer v2.18 [49].

## 3. Results

### 3.1. Dye Porous Kagomé Nanoarchitecture

**Figure 2:**
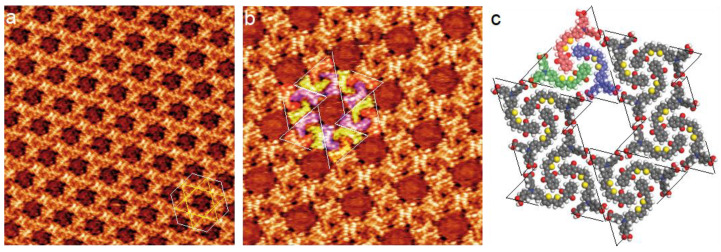
STM images of the self-assembly of compound-1 into a Kagomé nanoarchitecture with hexagonal cavities on graphite, (**a**) 38 × 38 nm^2^; (**b**) 20 × 20 nm^2^; V*_s_* = 0.6 V, I*_t_* = 450 pA. As a guide for the eyes, the hexagonal unit cell (white solid lines) and the Kagomé star (yellow solid lines) have been superimposed onto the STM image in (**a**). (**c**) Model of the Kagomé nanoarchitecture observed in (**a**,**b**). As a guide for the eyes, dashed white and black triangles highlighting triangular trimers have been superimposed onto the STM image in (**b**) and the model in (**c**), respectively.

The STM image in Figure 2a shows the self-assembly of compound-1 on the graphite surface after the deposition of a droplet of its solution. Molecules form a porous large-scale 2D Kagomé nanoarchitecture with hexagonal cavities. The network unit cell is a hexagon (white lines in Figure 2a) with 4.8 ± 0.1 nm unit cell constant (it should be noticed that the primitive network unit cell of this hexagonal nanoarchitecture is a lozenge with ~4.8 nm and ~4.8 nm unit cell constants and an angle of ~60° between the axes).

A high-resolution STM image of the molecular self-assembly is presented in Figure 2b. This image reveals that the cavities of the 2D nanoarchitecture result from the arrangement of molecular trimers in triangles (dashed white and black triangles in Figure 2b and Figure 2c, respectively). These trimers are composed of blue-, red- and green-colored molecules in Figure 2b,c. A model of the molecular arrangement is presented in Figure 2c [34]. Molecules do not adopt a straight conformation but an “L” conformation. This conformation maximizes intermolecular van der Waals interactions; there is no gap between the backbones of neighboring molecules. Neighboring trimers are rotated by 60° and are bonded through two double O···H−O hydrogen bonds between the carboxylic groups. This packing leads to the formation of hexagonal cavities (Diameter = ~2.0 nm) inside the organic network.

### 3.2. Coronene-Dye Guest–Host Nanoarchitecture

**Figure 3:**
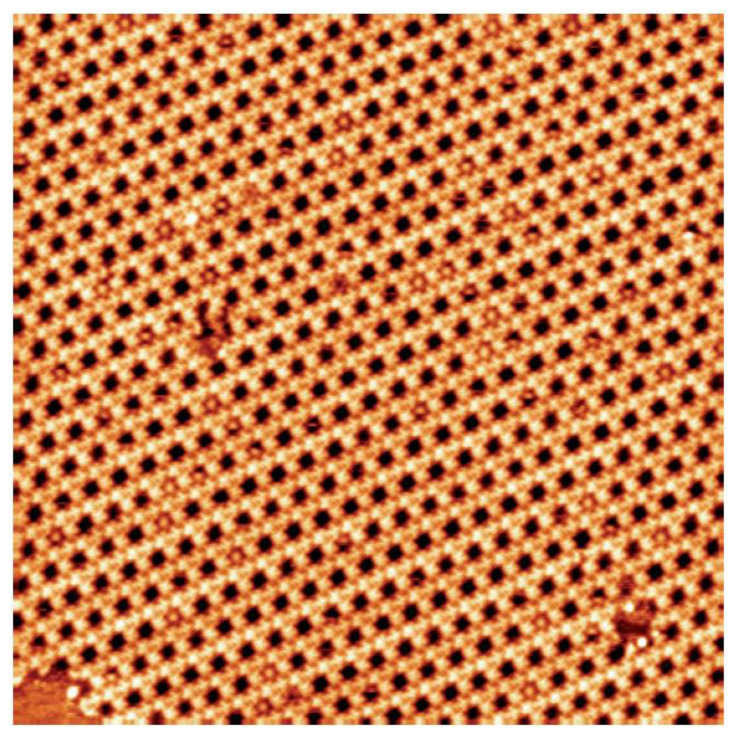
STM image of the dye Kagomé network after coronene deposition, 56 × 56 nm^2^; V*_s_* = 0.6 V, I*_t_* = 400 pA.

Coronene molecules at low concentration (2.0 × 10^−6^ M) are now deposited with the dye molecules on the graphite surface. The large scale STM image in Figure 3 shows that most of the Kagomé network cavities remain empty and only few cavities are filled with a coronene molecule. 

**Figure 4:**
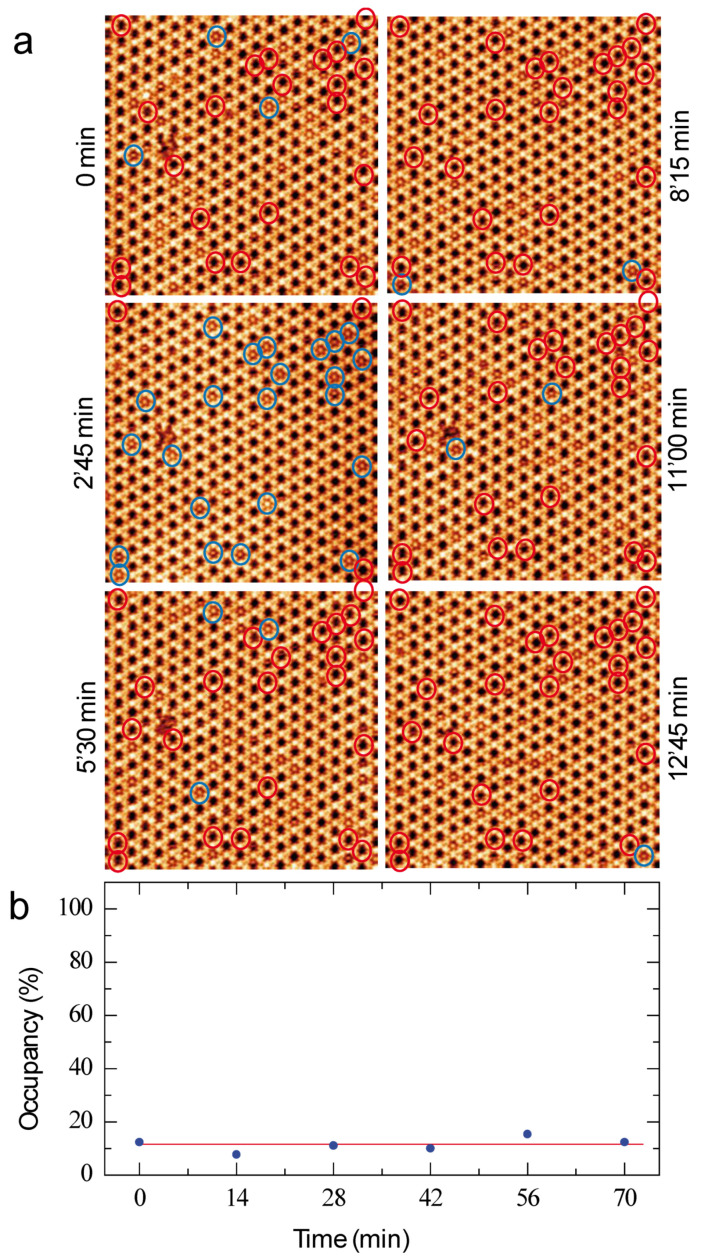
(**a**) Sequential STM images of the coronene adsorption into the organic dye Kagomé host-nanoarchitecture, 40 × 40 nm^2^; V*_s_* = 0.6 V, I*_t_* = 400 pA. The time between each image is 2 min and 45 s. (**b**) Evolution of the cavity occupancy of the host nanoarchitecture (percentage) with time (minutes). The red curve is a fit of the occupancy behavior.

Sequential STM images of the coronene-dye molecular assembly are now presented in Figure 4a. The time between two consecutive STM images is 2 min and 45 s. As a guide for the eyes, blue and red circles have been superimposed to twenty-six cavities of the dye Kagomé network in the six STM images in Figure 4a. The circles are blue when the cavity is filled with a coronene molecule, whereas the circles are red when the cavity is empty. STM images show that only four cavities are filled with a guest-molecule at the original time (0 min), whereas twenty-three cavities are filled 2′45 min later. After 12′45 min, only one of the highlighted cavities is occupied. The sequential STM images in Figure 4a thus reveal that the coronene molecules are constantly adsorbing and desorbing from the Kagomé network cavities. The time-dependent evolution of the cavity occupancy with coronene molecule is presented in Figure 4b: the cavity occupancy is about ~11% and is quite constant with time.

### 3.3. ZnPc-Dye Guest–Host Nanoarchitecture

In order to compare with the results obtained with coronene in Figure 4, ZnPc molecules (Figure 1b) at low concentration (2.0 × 10^−6^ M) are now deposited with the dye molecules on the graphite surface. 

**Figure 5:**
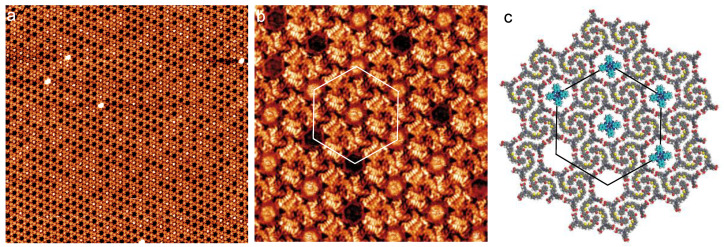
STM images of the organic layer after subsequent deposition of ZnPc with a low concentration (2.0 × 10^−6^ M), (**a**) 135 × 135 nm^2^, (**b**) 20 × 20 nm^2^; V*_s_* = 0.6 V, I*_t_* = 400 pA. (**c**) Model of the guest–host structure corresponding to the area with the superimposed white hexagon highlighted in (**b**).

A large-scale STM image of the organic layer recorded just after ZnPc deposition is presented in Figure 5a. This image is quite similar to those obtained with coronene molecules, Figure 4. Bright features corresponding to trapped ZnPc molecules in the compound-1 Kagomé nanoarchitecture are observed. Many cavities of the dye network are also empty. In Figure 5c is presented the molecular model of the guest–host structure highlighted by a yellow-colored area in the STM image in Figure 5b.

**Figure 6:**
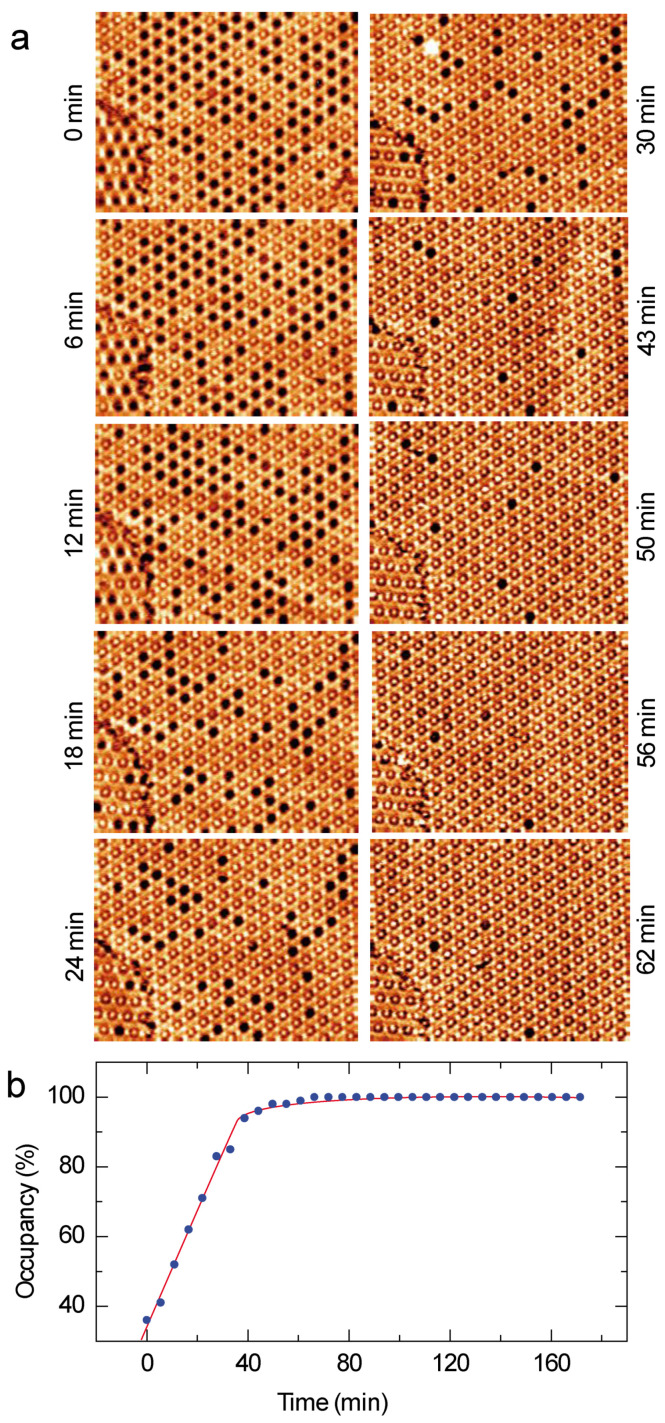
(**a**) Sequential STM images of the ZnPc adsorption into the organic host nanoarchitecture, 75 × 55 nm^2^; V*_s_* = 0.6 V, I*_t_* = 400 pA. (**b**) Evolution of the cavity occupancy of the host nanoarchitecture (percentage) with time (min). The red curve is a fit of the occupancy behavior.

**Figure 7:**
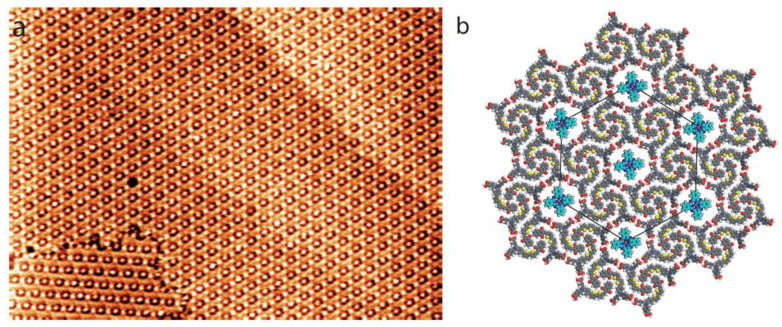
(**a**) STM image ZnPc-dye assembly after one hour, 120 × 85 nm^2^, V*_s_* = 0.6 V, I*_t_* = 400 pA. (**b**) Model of the ZnPc-dye guest–host structure.

In order to assess the dynamics of ZnPc trapping by the dye Kagomé network, the time-dependent evolution of the guest-host network is presented in Figure 6a. In contrast to coronene molecules (Figure 4), the sequential STM images reveal that cavity occupancy by ZnPc molecules of the network cavities increases with time. The evolution of the cavity occupancy with time is displayed in Figure 6b: cavity occupancy first evolves linearly. The top-left STM image in Figure 6a shows that only 40% of the Kagomé network cavities are filled with ZnPc. 55% of the cavities are filled after 33 min. The filling rate then slows once 95% of the network is occupied. It then takes 20 min for the 5% remaining empty cavities to be filled with a single ZnPc molecule. After one hour, nearly all the cavities of the Kagomé network are filled with one ZnPc molecule, Figure 7. It should be noticed that the empty cavities are gradually filled by ZnPc molecules and the desorption of the guest ZnPc molecules is rarely observed during the filling of the host structure cavities. This indicates that the space constraint is strong enough to hold the guest ZnPc molecules into the cavities of Kagomé network.

## 4. Discussion

The use of porous nanomaterial for molecular sensing relies on their ability to trap foreign molecular species at very low concentration.

STM shows that an extended porous 2D nanoarchitecture can be engineered taking advantage of the self-assembly of the dye molecule (Figure 2a). The molecules self-assemble on graphite surface into a Kagomé network with large cavities (Diameter = ~2.3 nm).

The versatility and trapping efficiency are then explored by depositing coronene and ZnPc molecules at low concentration (2.0 × 10^−6^ M) with the dye molecules. STM reveals that both coronene and ZnPc molecules can be trapped inside the Kagomé network cavities but the with different effectiveness.

The Figure 4 shows that only 11% of the Kagomé network cavities are filled with coronene molecules. This occupancy ratio does not evolve with time. These guest molecules are in addition temporary trapped in the host structure; they usually desorb from the surface few minutes after.

The behavior of the guest ZnPc molecules is drastically different. The Figure 6 shows that the number of trapped ZnPc molecules by the host structure linearly evolves with time, until 95% of the Kagomé cavities are occupied. After one hour, nearly all the cavities are filled. In comparison with coronene molecules, the desorption of the ZnPc from the cavities is rarely observed.

Solubility may affect molecular assembly. We selected 1-octanoic acid as the solvent because coronene and phtalocyanine molecules are known to be highly soluble in it. This high solubility may explain why no pure coronene or phthalocyanine networks are observed on the surface. Coronene in host cavities are desorbing from the surface, whereas ZnPc are not. This shows that the adsorption efficiency of ZnPc is higher than the one of coronene under the same concentration. Therefore, the size of guest molecule relative to the host-porous size is the critical factor for their trapping efficiency. Only intermolecular interactions with the host structure allows immobilizing these guest molecules on the surface. The experimental STM observation highlights that the dye Kagomé network is a more efficient porous structure to trap ZnPc molecules at low concentration. The Kagomé network cavities are ~2.3 nm large, whereas the coronene and ZnPc molecules have a diameter of 1.0 nm and 1.4 nm, respectively. The large size discrepancy between the cavity dimensions and the coronene molecule explain why the coronene molecule are desorbing from the surface due to the weak steric constraints between the coronene and the dye network. In contrast, as the ZnPc molecules have larger dimensions, the steric constraints between the guest molecules and the host structure are large enough to keep the ZnPc molecules trapped in the dye network. The most optimized space matching of ZnPc with the size of the Kagomé network cavities appears therefore to be the main driving force stabilizing the guest-host nanostructure (i.e., trapping permanently the ZnPc molecules in the host structure; an effect which is not observed with smaller coronene molecules).

## 5. Conclusions

In this paper, we investigated the trapping of coronene and ZnPc molecules by a porous 2D self-assembled nanoarchitecture of a push–pull dye on a graphite surface. The dye molecules form a hexagonal Kagomé network with large empty cavities. STM shows that this host structure can be used to trap coronene and ZnPc molecules in its cavities at a low concentration. The coronene molecules are, however, desorbing and the host structure is never saturated with guest molecules at low concentration, whereas almost all the cavities of the host structure are filled with ZnPc molecules after a few hours. The geometry of the 2D nanoarchitecture opens up new opportunities for trapping specific foreign molecules, especially phthalocyanine-based compounds, for engineering novel organic nanomaterials for applications in molecular sensing and organic electronics [50,51,52]. This structure is a model system for investigating local electronic coupling [53] and charge transfer [54] between foreign organic species. Future experimental research will also focus on exploring the ability of the host structure to trap functionalized guest molecules with different shape and substituents to trigger intermolecular interactions other than van der Waals ones.

## Data Availability

Data are contained within the article.
